# Effects of Cold Jet Atmospheric Pressure Plasma on the Structural Characteristics and Immunoreactivity of Celiac-Toxic Peptides and Wheat Storage Proteins

**DOI:** 10.3390/ijms21031012

**Published:** 2020-02-04

**Authors:** Fusheng Sun, Xiaoxue Xie, Yufan Zhang, Jiangwei Duan, Mingyu Ma, Yaqiong Wang, Ding Qiu, Xinpei Lu, Guangxiao Yang, Guangyuan He

**Affiliations:** 1 The Key Laboratory of Molecular Biophysics of Chinese Ministry of Education, The Genetic Engineering International Cooperation Base of Chinese Ministry of Science and Technology, College of Life Science and Technology, Huazhong University of Science and Technology, Wuhan 430074, China; fufu4567@126.com (F.S.); xiexiaoxue_Cecilia@163.com (X.X.); zhangyufan1108@126.com (Y.Z.); wangyaqiongyou@126.com (Y.W.); qiuding1989@outlook.com (D.Q.); 2 State Key Laboratory of Advanced Electromagnetic Engineering and Technology, School of Electrical and Electronic Engineering, Huazhong University of Science and Technology, Wuhan 430074, China; duanjiangwei@foxmail.com (J.D.); mmyhfut@163.com (M.M.); luxinpei@hotmail.com (X.L.)

**Keywords:** cold jet atmospheric pressure plasma, reactive oxygen and nitrogen species, backbone cleavage, hydroxylation, carbonyl formation

## Abstract

The present research reported the effects of structural properties and immunoreactivity of celiac-toxic peptides and wheat storage proteins modified by cold jet atmospheric pressure (CJAP) plasma. It could generate numerous high-energy excited atoms, photons, electrons, and reactive oxygen and nitrogen species, including O_3_, H_2_O_2_, •OH, NO_2_^−^ and NO_3_^−^ etc., to modify two model peptides and wheat storage proteins. The Orbitrap HR-LC-MS/MS was utilized to identify and quantify CJAP plasma-modified model peptide products. Backbone cleavage of QQPFP and PQPQLPY at specific proline and glutamine residues, accompanied by hydroxylation at the aromatic ring of phenylalanine and tyrosine residues, contributed to the reduction and modification of celiac-toxic peptides. Apart from fragmentation, oxidation, and agglomeration states were evaluated, including carbonyl formation and the decline of γ-gliadin. The immunoreactivity of gliadin extract declined over time, demonstrating a significant decrease by 51.95% after 60 min of CJAP plasma treatment in vitro. The CJAP plasma could initiate depolymerization of gluten polymer, thereby reducing the amounts of large-sized polymers. In conclusion, CJAP plasma could be employed as a potential technique in the modification and reduction of celiac-toxic peptides and wheat storage proteins.

## 1. Introduction

Gluten proteins in wheat (*Triticum aestivum* L.) are a type of storage protein-containing gliadins and glutenins, which are responsible for the rheological and viscoelastic properties of wheat dough [1]. Gluten proteins contain abundant proline (Pro) and glutamine (Gln) residues, which can cause high resistance of gluten peptides to be hydrolyzed, a property that contributes to the gluten-related immunogenic nature of celiac disease (CD) patients [2]. The degradation of gluten proteins can decrease their immunological and toxic properties. To date, some biological, chemical, and physical approaches have been applied to reduce toxicity of these proteins to patients with CD. Biological strategies include downregulation of gliadin expression by CRISPR-Cas9 to produce low-gluten wheat lines with an average reduction of 66.7% and 61.7% with regard to R5 and G12 antibody reactivity [3]. For biochemical processing, usage of food-grade microbial transglutaminase (mTG) with lysine ethyl ester is able to modify gliadin peptides, which inhibits the ability of gluten to trigger the specific human immune response in vitro [4]. Huang et al. (2017) found a metal-catalyzed oxidation system can cause the modification and elimination of hordein, leading to the decrease of immunoreactivity of hordein [5].

Although enzymatic and chemical methods possess high efficiency in the modification of macromolecular compound, shortcomings of these techniques are concerned in points of higher cost, wastewater pollution and food safety etc. Physical techniques could make up for these deficiencies of enzymatic and chemical modifications [6]. The high temperature treatment modifies protein through denaturation and formation of inter/intramolecular bonds, which consequently resulted in specific epitopes unrecognizable by antibody [7]. Also, it has been reported that ozone, excited atoms, ions and electrons can be considered as efficient species because they can act as strong oxidizing agents for the object without leaving any residues [8]. According to this principle, cold plasma is regarded as an emerging non-thermal technique to modify the characteristics of gluten in wheat flour [9]. Cold plasma, regarded as the source of reactive oxygen and nitrogen species (RONS), is an ionized gas consisting of a variety of types of active species, such as singlet oxygen, ozone, excited molecular nitrogen, and other electrons or ions [10]. Exploitation of plasma has been widely researched in nonfood areas, such as the inactivation of bacteria, wound healing, tooth bleaching, and cancer therapy [11,12,13,14]. However, whether it can modify wheat gluten proteins to reduce the toxicity of celiac disease has rarely been investigated. 

At present, the common method for quantification of food prolamins is based on antibodies against the epitopes Gln-Gln-Pro-Phe-Pro (QQPFP) and Pro-Gln-Pro-Gln-Leu-Pro-Tyr (PQPQLPY) [5]. Therefore, two model celiac-toxic peptides, QQPFP-repetitive domain in the γ-type, α-type and ω-type of prolamins in barley, rye and wheat, PQPQLPY-repetitive sequence in 33-mer peptide, were selected for plasma modification in our study [15]. Arentz-Hansen et al. (2002) previously stated that the toxicity mechanism of QQPFP sequence to CD patients may be due to its involvement in triggering T-cell activation after the deamidation of specific glutamine residues [16]. Furthermore, three previously identified celiac-specific T-cell epitopes play an important role in T-cell proliferation, namely, PQPQLPYPQ, PFPQPQLPY and PYPQPQLPY, all of which contain heptapeptide PQPQLPY [5]. In addition, exposure of Pro, Gln and phenylalanine (Phe) provides an important recognition site for G12 or R5 antibody, or stimulates the T-cell response [17]. Consequently, wheat gliadins in food containing those epitopes should be modified before their consumption in CD patients [18].

The objective of this study was to investigate the understanding of impacts of CJAP plasma on the structural properties and immunoreactivity of celiac-toxic peptides and wheat storage proteins in vitro. The effects of fragmentation at Pro and Gln and hydroxylation at Phe and tyrosine (Tyr) on celiac peptides-QQPFP and PQPQLPY, modifications and depolymerization behavior of gluten proteins, along with their immunoreactivity against R5 antibody after CJAP plasma treatment, were examined in this research.

## 2. Results

### 2.1. Experiment Setup, Discharge, Current and Optical Emission Spectrum (OES) Measurements

**Figure 1:**
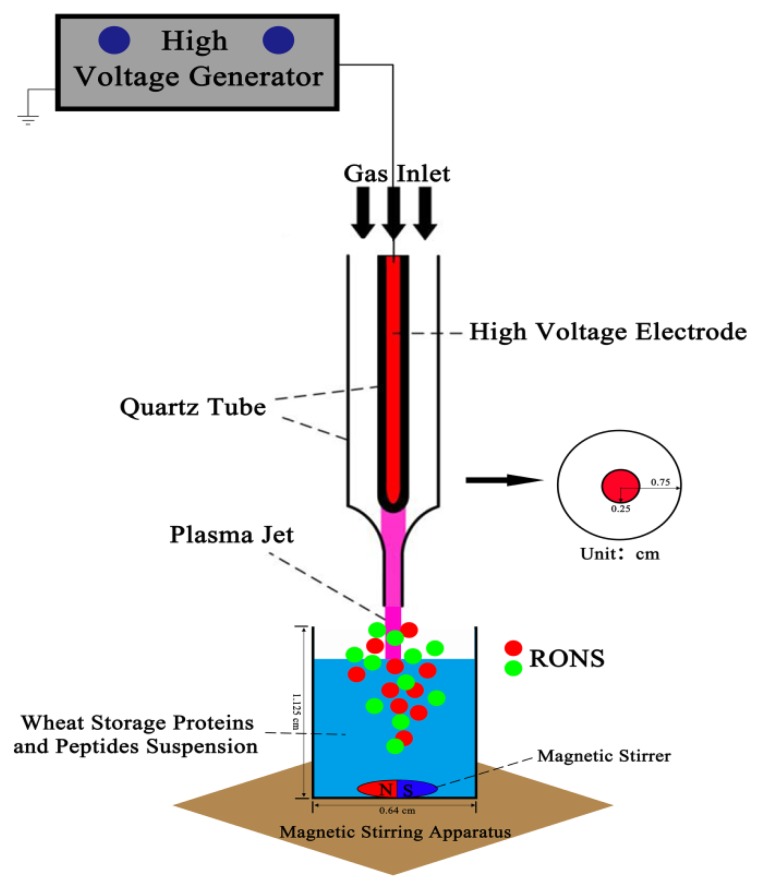
Schematic diagram of the plasma setup. The model peptides and gluten proteins in the 96-well plate were treated using a cold jet atmospheric pressure (CJAP) plasma jet.

The schematic diagram of experiment setup is shown in Figure 1. The celiac-toxic peptides and wheat storage protein samples in 96-well plate (200 μL per sample) were modified by the special CJAP plasma. The discharge gas consisted of helium at a flow rate of 2 L/min and air at a flow rate of 10 mL/min. The distance from the surface of slurry to jet nozzle was 10 mm. The samples were modified by CJAP plasma at different time points (0, 5, 10, 20, 30, and 60 min).

**Figure 2:**
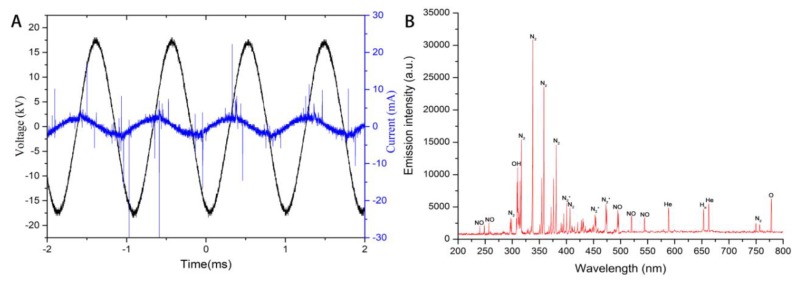
The waveforms of applied voltage and current (**A**) and the optical emissions spectra (OES) of the CJAP plasma jet (**B**).

The wave-forms of set voltage and single current of CJAP plasma were shown in Figure 2A. The peak-to-peak set voltage was 35 kV and frequency was 1 kHz. The OES of plasma were presented in Figure 2B, which were detected using an Ocean optics spectrometer. The emission lines of N_2_, N_2_^+^, NO, •OH, O, and He can be found when the discharge gas is air [19].

### 2.2. Generation of Several RONS Induced by CJAP Plasma

In plasma processes, active species such as radicals and ions induced by the plasma play a dominant role. To study the effects of various RONS on plasma modification of two model peptides and gliadin, the contents of H_2_O_2_, OH radicals, O_3_ and NO_2_^−^ + NO_3_^−^ were determined in this part.

**Figure 3:**
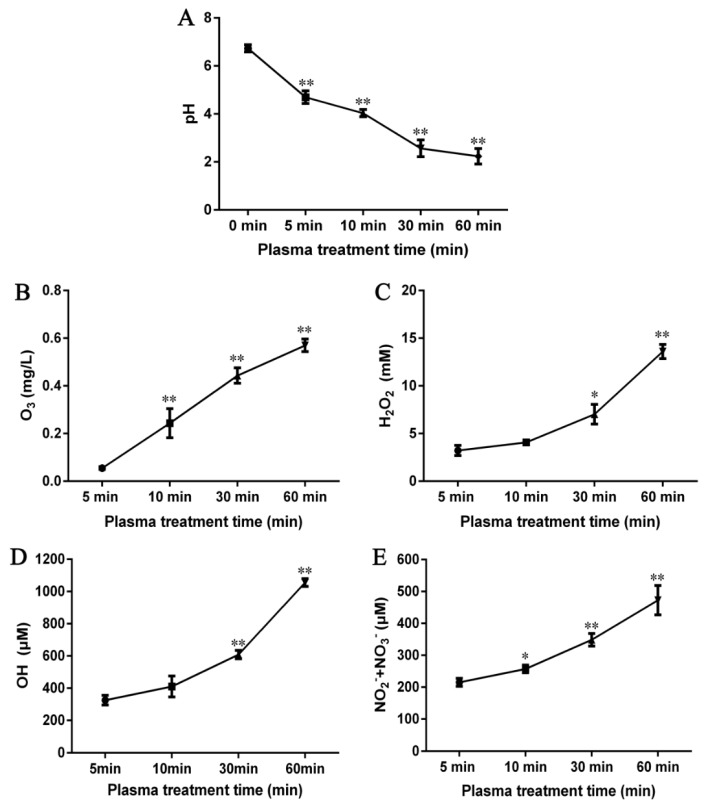
The pH value (**A**) and concentrations of O_3_ (**B**), H_2_O_2_ (**C**), •OH (**D**), and total NO_2_^−^ and NO_3_^−^ (**E**) in CJAP plasma with treatment times of 5 min, 10 min, 30 min, and 60 min. Data are presented as means ± SE (*n* = 3) in the pH and reactive oxygen and nitrogen species (RONS) detection assays. Asterisks represent statistically significant differences from 5 min (* *p* < 0.05, ** *p* < 0.01).

In order to understand the acidity and alkalinity of samples after treating the plasma, the pH value was determined. The pH value results showed a longer plasma treatment time correlated with a greater decline in pH value. From Figure 3A, it can be seen that the pH value decreased from 6.7 at 0 min to 2.2 after 60 min.

Ozone is reported to selectively react with substrates by electrophilic attack and cycloaddition, while decomposing to produce OH radicals as another non-selective oxidant [20,21]. For a better understanding of the changes in ozone content following plasma initiation, results of the O_3_ content were measured (Figure 3B), which presented a time-dependent increase, with significant increase (*p <* 0.01) at 10 min over control. It was found that the concentration of O_3_ of the treated samples are 0.06 mg/L, 0.24 mg/L, 0.45 mg/L, and 0.58 mg/L at 5 min, 10 min, 30 min, and 60 min of plasma treatment, respectively.

H_2_O_2_ was considered to be another oxidizing agent and it was selected to be detected in this paper [22]. From Figure 3C, increase in H_2_O_2_ concentration was dependent on treatment time within 60 min, with the biggest increase from 30 to 60 min. We can see that the H_2_O_2_ content of the treated samples expanded from 3.23 mM at 5 min to 13.60 mM at 60 min.

For the most reactive form of ROS, OH radicals can undergo various types of reactions with amino acid side chains and peptide backbone [23]. Figure 3D shows that the pattern of change in •OH content was similar to that of H_2_O_2_. A significant (*p <* 0.01) increase occurred at 30 min, followed by a further increase at 60 min (*p <* 0.01). The •OH content was 325.53 μM at 5 min, 410.31 μM at 10 min, 608.30 μM at 30 min and 1055.10 μM at 60 min.

High oxidation features could also be possessed by reactive nitrogen species. To demonstrate whether nitrogen oxides were generated during plasma treatment to aid in modification and oxidation of proteins and peptides, the total NO_2_^−^ and NO_3_^−^ contents were measured. Figure 3E shows that the total contents of NO_2_^−^ and NO_3_^−^ increased rapidly to 215.21 μM at 5 min and then further increased at 60 min, reaching 472.83 μM.

### 2.3. Oxidation and Hydroxylation Gives Rise to P1~P7 after CJAP Plasma Treatment

Under non-plasma treatment conditions, the major peaks on the chromatograms were two original peptides, QQPFP (Supplementary Figure S1A) and PQPQLPY (Supplementary Figure S1B). After CJAP plasma modification, the peak intensities of two model peptides decreased and were almost undetectable after 60 min of treatment. With plasma processing, several new peaks appeared, mainly P1~P7. Fragmentation continued with continued treatment, but after 60 min, most of peaks had disappeared and less peptide was detected in columns.

### 2.4. Modification of Specific Pro, Gln, Phe, and Tyr Residues Leads to Degradation of the Model Peptides

**Figure 4:**
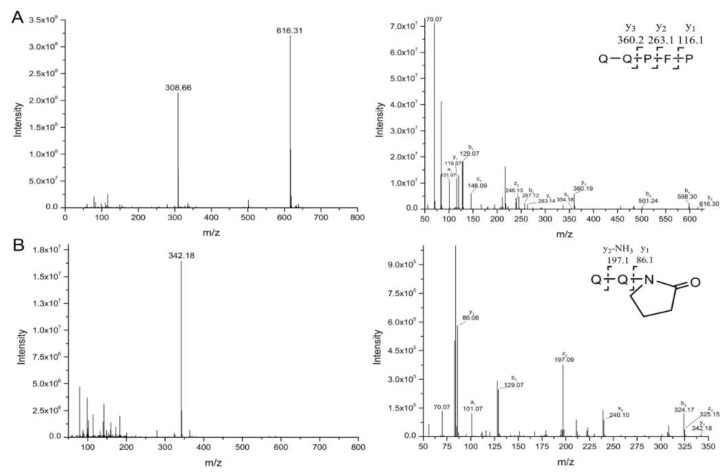

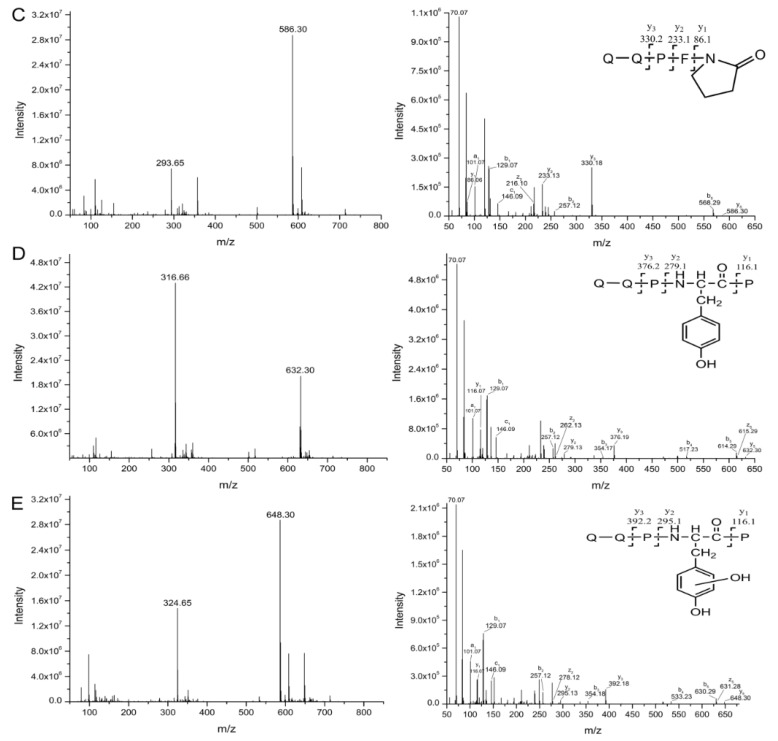
Full-scan mass spectrometry (MS) and representative MS/MS spectra of QQPFP and its products (P1~P4) during plasma treatment, showing evidence for •OH-mediated modification. In each panel, the left spectrum shows the full scan, while the right spectrum shows the fragment ions. The b and y ions originated from preferential cleavage at the N-terminus and C-terminus of specific amino acid residues during collision-induced dissociation, respectively. (**A**) Original peptide (616.3 *m*/*z*); (**B**) product 1 (342.2 *m*/*z*); (**C**) product 2 (586.3 *m*/*z*); (**D**) product 3 (632.3 *m*/*z*); (**E**) product 4 (648.3 *m*/*z*).

**Figure 5:**
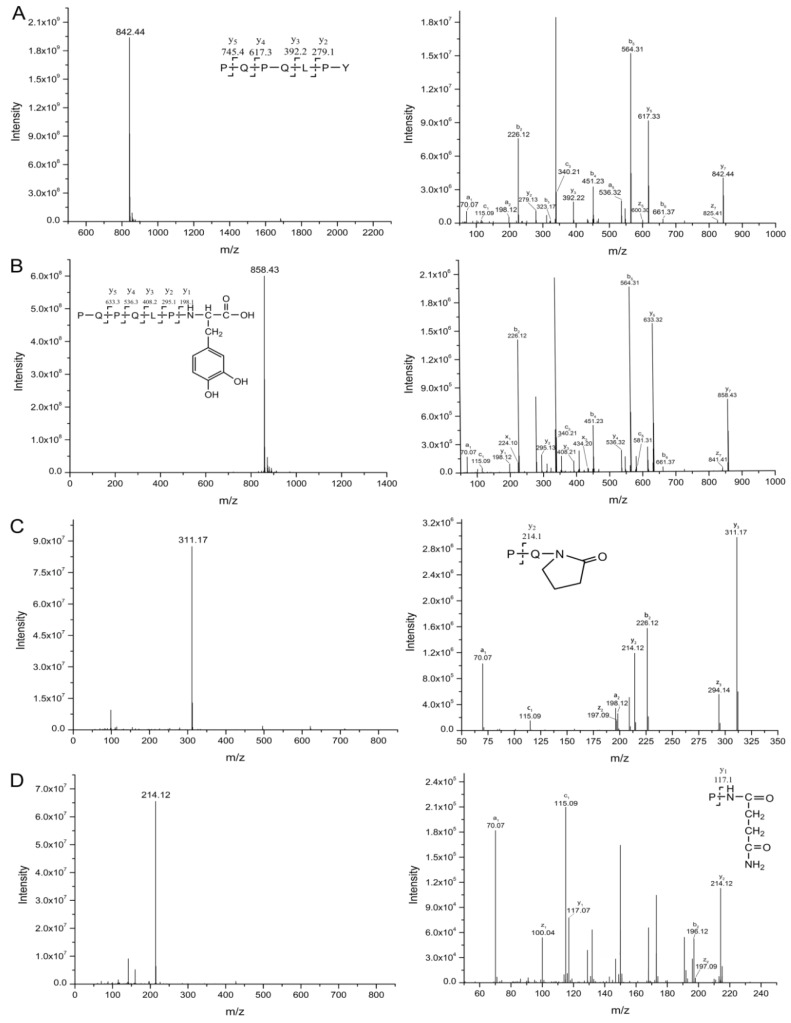
Full-scan MS and representative MS/MS spectra of PQPQLPY and its products (P5~P7) during plasma treatment, showing evidence for •OH-mediated modification. In each panel, the left spectrum shows the full scan, while the right spectrum shows the fragment ions. The b and y ions originated from preferential cleavage at the N-terminus and C-terminus of specific amino acid residues during collision-induced dissociation, respectively. (**A**) Original peptide (842.4 *m*/*z*); (**B**) product 5 (858.4 *m*/*z*); (**C**) product 6 (311.7 *m*/*z*); (**D**) product 7 (214.1 *m*/*z*).

The MS/MS spectra of QQPFP (616.27 *m*/*z*) and PQPQLPY (842.44 *m*/*z*) exhibited several fragmentation ions relating to b, x, y and z ions in the process of collision-induced dissociation (Figure 4A and Figure 5A). For oxidation products, including P1, P2, and P6, there was a mass shift of −30 *m*/*z* for several y and b ions, corresponding to the formation of 2-pyrolidone at specific Pro residues on peptides with the loss of –COOH (−45 *m*/*z*) and subsequent addition of O (+16 *m*/*z*) linked to a double bond (−1 *m*/*z*) (Figure 4B,C and Figure 5C). In their hydroxylation products, namely, P3 and P5, the y2, y3, and y5 (only for P5) fragment ions showed an increase of 16 *m*/*z*, relevant to the addition of a hydroxyl group on the aromatic ring of peptides (Figure 4D and Figure 5B). Similarly, P4 displayed a mass shift of +32 *m*/*z* for the y1, y2 and y3 fragment ions, suggesting that two hydroxyl groups were added to the benzene ring of specific pentapeptide (Figure 4E). 

**Figure 6:**
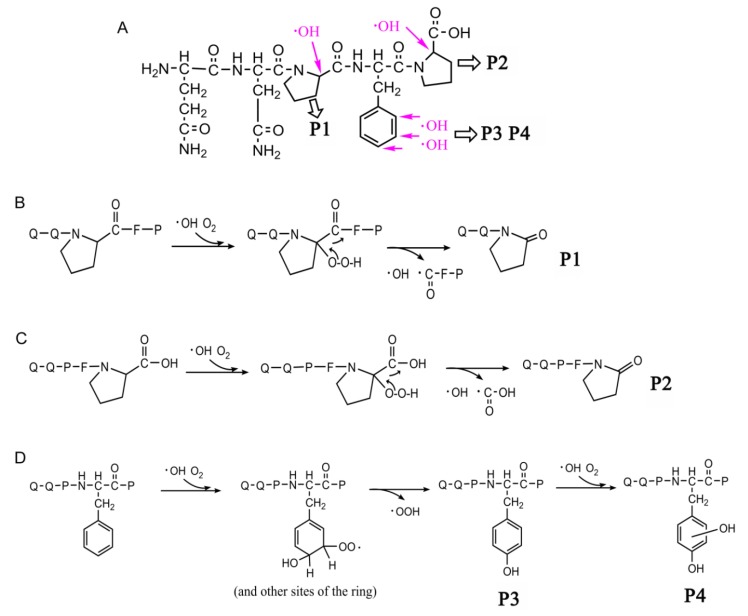
Structures of the celiac-toxic peptide QQPFP and its CJAP plasma-modified products referred to in this research (P1~P4), which were detected by Orbitrap HR-LC-MS/MS. (**A**) Structural changes in QQPFP during CJAP plasma treatment. The arrow shows the sites that were attacked at the intermediate region and C-terminus of the specific pentapeptide in this study. (**B**) The reaction pathway of P1 formation involved in •OH attack at the Pro residue in the intermediate region. (**C**) The reaction pathway of P2 formation associated with •OH attack at the Pro residue in the C-terminus. (**D**) P3 and P4 were formed through the reaction involved in •OH attack at the benzene ring of Phe, followed by a reaction with O_2_ and, subsequently, elimination of •OOH.

**Figure 7:**
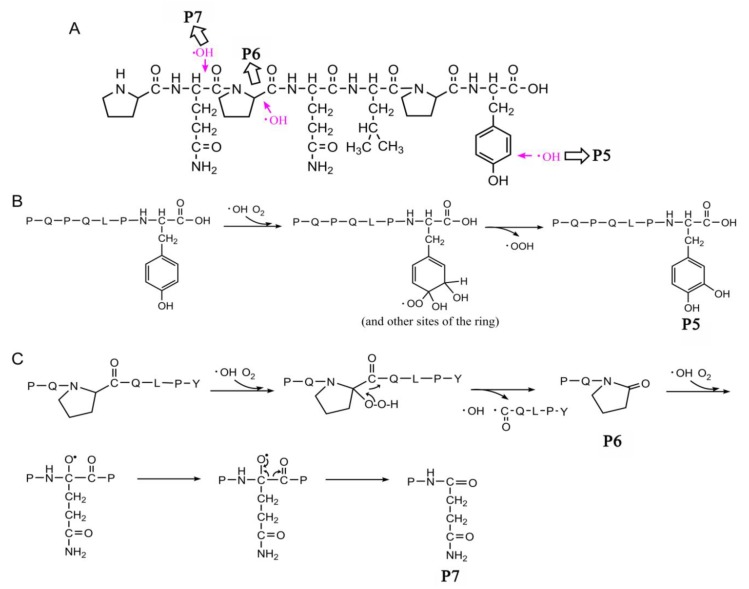
Structures of the celiac-toxic peptide PQPQLPY and its CJAP plasma-modified fragmentation products referred to in this research (P5~P7), which were detected by Orbitrap HR-LC-MS/MS. (**A**) Structural changes in PQPQLPY during CJAP plasma treatment. The arrow shows the sites attacked in the intermediate region and C-terminus of the specific pentapeptide in this study. (**B**) P5 was formed through the reaction involving •OH attack at the benzene ring of Tyr, followed by a reaction with O_2_ and subsequent elimination of •OOH to form a mixture of DOPA stereoisomers. (**C**) The reaction pathway of P6 and P7 formation associated with •OH attack at the Pro residue and the formation of an alkoxy group on the α-carbon of Gln.

After plasma processing of original peptides, the Orbitrap HR-LC-MS/MS distinguished products P1, P2, P6 (Figure 6B,C and Figure 7C), in which Pro residues were attacked, as well as P3, P4 and P5 (Figure 6D and Figure 7B), with the addition of hydroxyl groups on phenyl ring of Phe, leading to one or two +16-Da mass shifts. QQPFP gave rise to CJAP plasma-induced oxidation products at *m*/*z* 342.12 (P1) and *m*/*z* 586.30 (P2), as well as hydroxylation products at *m*/*z* 632.30 (P3, hydroxyphenylalanine) and *m*/*z* 648.30 (P4, dihydrophenelalanine, DOPA), while PQPQLPY generated products at *m*/*z* 858.43 (P5), *m*/*z* 311.17 (P6) and *m*/*z* 214.11 (P7) (Figure 4 and Figure 5).

As shown in Supplementary Figure S2A, plasma treatment exceeding 10 min resulted in detectable Pro fragmentation and hydroxylation of Phe in the peptide. After 10 min of plasma treatment, 40.21% of QQPFP and 66.73% of PQPQLPY remained intact, and further fragmentation was detected after a longer period of treatment, causing a decrease in QQPFP and PQPQLPY, leaving 1.43% and 15.03% at 30 min, and 0.16% and 0.08% at 60 min, respectively. Moreover, a shorter fragment, P7, was also detected and exhibited a tendency to increase over time (Supplementary Figure S2C). Extract ion chromatograms (EIC) opting for certain fragmentation ions were utilized to certify the intensity changes in P3, P4, and P5, which contained the different stereoisomers (Supplementary Figure S3).

### 2.5. Agglomeration State of Wheat Gliadins Analyzed by SDS-PAGE

To display size changes in an observable way, gliadin proteins with and without plasma treatment were separated on the 12% SDS-PAGE. Comparing plasma-treated with untreated samples (Supplementary Figure S4), a decrease in intensity of stripe S1 was observed at the bottom of polyacrylamide gel. Additionally, another visible decrease in intensity of stripes S2 and S3 could also be seen in the plasma-treated samples. These observations demonstrated larger-sized proteins might undergo fragmentation to generate smaller-sized proteins under plasma conditions due to the depolymerization behavior of wheat gliadins during their exposure to the CJAP plasma.

### 2.6. Decrease in Quantity of Wheat Gliadins Treated with CJAP Plasma

**Figure 8:**
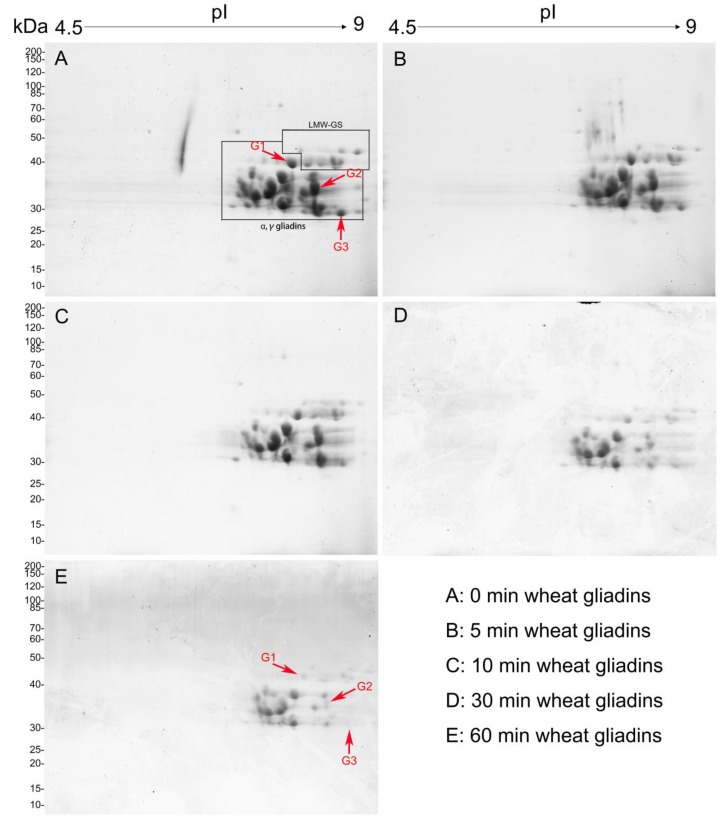
2-DE detection of protein species in native and CJAP plasma-modified wheat gliadins. The major groups of wheat gliadins are indicated. The molecular weight is indicated on the vertical axis, and isoelectric point is shown on the horizontal axis. The A-E are the wheat gliadin samples treated with CJAP plasma for 0, 5, 10, 30 and 60 min, respectively.

To explore the CJAP plasma-treated specific gliadins changes in more detail, the 2-DE gel of gliadin extract is shown in Figure 8, which revealed that some protein spots disappeared or were reduced in quantity with longer CJAP plasma treatment. By comparison with the untreated sample, particular attention was directed toward three protein spots (G1, G2, and G3) with obviously reduced intensity in the treated samples. They were subjected to LC-MS/MS to clarify the type of gliadin. To clarify the type of gliadin susceptible to CJAP plasma, those three protein spots arrowed in Figure 8 were subjected to LC-MS/MS experiment for further detection.

### 2.7. LC-MS Identification of Proteins in 2-DE Spots

Proteins in 2-DE spots indicated by arrows were identified via LC-MS. Table 1 shows that γ-type gliadins were detected as the predominant protein in those three spots. In these spots, the predominant protein of G1 was a traditional gamma gliadin with an MW of 37,564 Daltons and pI of 8.51. An additional two protein spots (G2 and G3) with an MW of 34,502 and 33,067 Daltons with corresponding pI of 8.57 and 8.88 were also detected as gamma gliadin. More detailed information regarding these spots is presented in Table 1 below.

### 2.8. Increment of Carbonyl Groups in Plasma-Treated Wheat Gliadins

**Figure 9:**
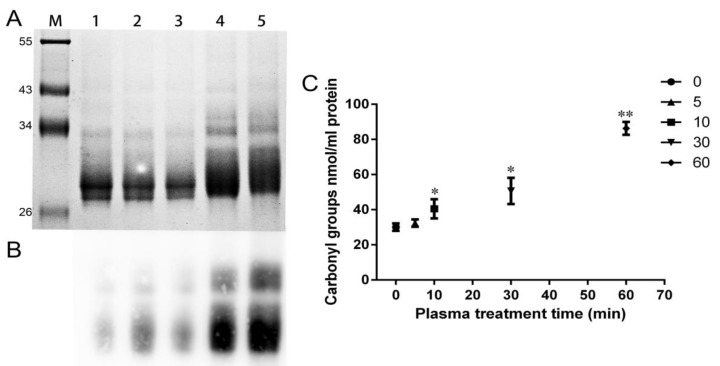
Qualitative and quantitative analysis of carbonyl groups of the wheat gliadin samples treated with CJAP plasma for 0 min, 5 min, 10 min, 30 min and 60 min, respectively. (**A**) SDS-PAGE gel stained with CBB. M: Protein molar mass standard, lane 1: 0 min, lane 2: 5 min, lane 3: 10 min, lane 4: 30 min, lane 5: 60 min. (**B**) Western blot of carbonyl groups using biotin-hydrazide. (**C**) Quantification of carbonyl groups using the 4-dinitrophenyl-hydrazine (DNPH) method. Data are presented as means ± SE (*n* = 3) in the quantitative detection assays. Asterisks represent statistically significant differences from 0 min (* *p* < 0.05, ** *p* < 0.01).

Carbonyl groups are frequently used as biomarkers to demonstrate the protein oxidation level in biological samples [24]. As shown in Figure 9A, the separation gel presented new aggregates resulting from CJAP plasma-induced oxidation of wheat gliadins. Although carbonyl groups were formed in both plasma-treated and untreated samples, dissimilar blot intensities were observed in the samples, with the strongest intensity apparent at 30 and 60 min (Figure 9B). The quantification results (Figure 9C) revealed an increase in carbonyl groups with plasma treatment from 32.36 nmol/mL at 5 min to 86.30 nmol/mL at 60 min, with a significant increase (*p* < 0.05) first appearing at 10 min and a highly significant increase (*p* < 0.01) at 60 min compared with the untreated sample. These results showed that the CJAP plasma-treated wheat gliadins formed more carbonyl groups.

### 2.9. Immunological Activity Evaluation of Wheat Gliadins in ELISA

**Figure 10:**
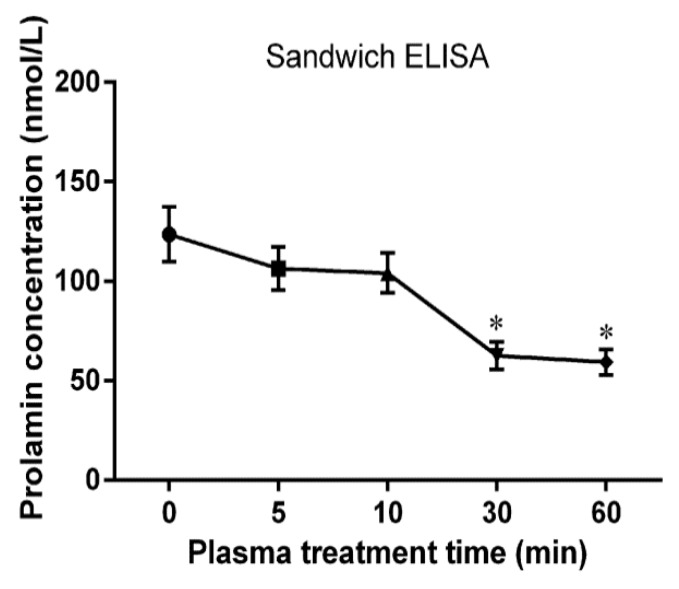
Sandwich ELISA of native and CJAP plasma-modified wheat gliadins. Data are presented as means ± SE (*n* = 3) in the ELISA system. Asterisks represent statistically significant differences from 0 min (* *p* < 0.05).

The R5 antibody can specifically recognize the epitope QQPFP, which exists in nearly all parts of gliadins [5]. The immunological activities of CJAP plasma-treated wheat gliadins were determined by ELISA. As shown in Figure 10, R5 antibody immunoreactivity in all treated samples exposed for different durations of plasma treatment were lower than in the untreated sample. After 5 min of plasma treatment, the prolamin concentration declined to 86.12% of its original level, and thereafter 84.23% of its initial content remained after 10 min of treatment. The lowest remaining prolamin concentration, at 48.05% of its initial level, was detected in samples treated with plasma for 60 min (*p <* 0.05). The decline in prolamin content implied that the epitopes for R5 recognition were modified by plasma treatment, which caused a reduction of immunological activity of wheat gliadins in vitro.

### 2.10. Size Distribution Change Analysis of Gluten Proteins before and after CJAP Plasma Treatment

**Figure 11:**
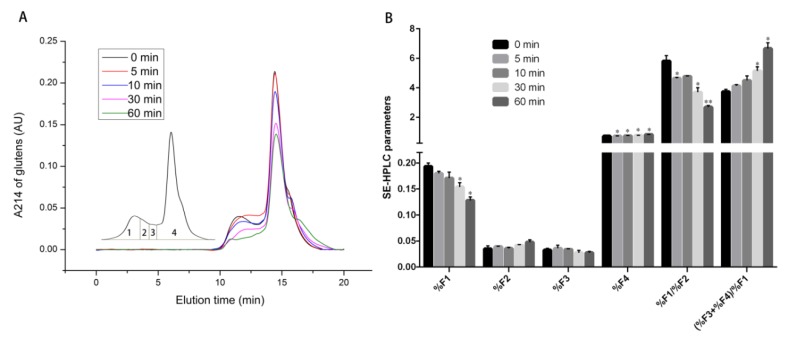
The influence of CJAP plasma on the aggregate formation of wheat gluten proteins. (**A**) SE-HPLC chromatogram curve of native and CJAP plasma-modified wheat gluten proteins. The inset refers to the area division of the wheat storage protein distribution, which corresponded to large-sized polymers (F1), medium-sized polymers (F2), monomeric and oligomeric proteins (F3) and monomeric gliadins and non-gluten proteins (F4). (**B**) with corresponding comparisons of six parameters (%F1, %F2, %F3, %F4, %F1/%F2, and (%F3 + %F4)/%F1) analyzed statistically. Data are presented as means ± SE (*n* = 3) in the assays. Asterisks represent statistically significant differences from 0 min (* *p* < 0.05, ** *p* < 0.01).

SE-HPLC was a brilliant method for comparison of the molecular size distribution of native and plasma-treated gluten proteins by quantisation of the elution profiles and the size fractionation of those proteins with plasma treatment gave a good intimation of the degree of protein oxidation and modification. In the presence of RONS generated by CJAP plasma, an explicit decline of the peak height of SE-HPLC profiles was observed in the treated samples compared with untreated sample (Figure 11A). As shown in Figure 11B, lower values of F1% were observed at 5 min and 10 min compared with the untreated sample, with the biggest decrease observed at 60 min (*p* < 0.05). Besides, the ratios of F1%/F2% of CJAP plasma-treated samples gradually decreased and the most significant decline was achieved at 60 min (*p* < 0.01). The sample treated for 30 min had significantly higher ratios of (F3% + F4%)/F1% and a further increase appeared at 60 min (*p* < 0.05). These results implied that there was a time-dependent decrease in the amounts of large-sized polymers in plasma-treated samples when compared to the untreated one, suggesting that depolymerization of macropolymers probably occurred during plasma treatment.

## 3. Discussion

In this context, an assortment of reactive oxygen and nitrogen species such as O_3_, •OH, H_2_O_2_, NO_2_^−^, and NO_3_^−^ generated during plasma treatment entailed that samples were subjected to an environment with high possibility of oxidation and modification. Moreover, the immunoreactivity of gliadin in wheat was successfully attenuated via CJAP-plasma modification, with oxidation and hydroxylation being of the major importance. Meanwhile, an Orbitrap HR-LC-MS/MS method was utilized to demonstrate the degree of oxidation at specific Pro and Gln and hydroxylation at Phe and Tyr through analyzing the products originated from plasma-treated peptides, QQPFP, and PQPQLPY.

### 3.1. CJAP Plasma can Generate a Variety of RONS

A diversity of RONS such as O_3_, •OH, H_2_O_2_, NO_2_^−^ and NO_3_^−^ generated during CJAP-plasma treatment, indicating samples were subjected to an environment with a high possibility of oxidation and hydroxylation modification. RONS is in a dynamic process of mutual transformation. The OH radicals were found to be of great significance for oxidation and hydroxylation reactions. There are mainly two kinds of reaction pathways leading to the generation of OH radicals, which may explain the gradual increase of •OH density during CJAP-plasma treatment [25,26]. One is the dissociation of radicals and metastables (Reactions 1 and 2), and the other is the electron dissociation of H_2_O (Reaction 3). Furthermore, it is well-documented that O_3_ can preferentially produce in DBD, and O_3_ mainly emerged from the reaction (Reaction 4) between the dissolved atomic oxygen and molecular oxygen [27,28].

e + O_2_ → O(^3^P) + O(^1^D) (1)

O(^1^D) + H_2_O → 2OH (2)

e^−^ + H_2_O → OH + H + e^−^ (3)

O + O_2_ ↔ O_3_ (4)

According to Liu et al. (2016), the interaction (Reaction 5) between HO_2_ and HO_3_ is responsible for H_2_O_2_ generation [28]. It is worth mentioning that highly reactive OH radicals with a high density within a small diffusion length should be balanced via the recombination reaction (Reaction 6), which also contributes to the increase in H_2_O_2_.

HO_2_ + HO_3_ → H_2_O_2_ + O_2_ (5)

OH + OH → H_2_O_2_ (6)

During the course of plasma treatment, various nitrogen oxides such as NO, NO_2_^−^ and NO_3_^−^ were detected, which presumably accounted for the decrease in pH value and increase in the density of NO_2_^−^ and NO_3_^−^ in our experiment [29].

### 3.2. CJAP Plasma-Induced Oxidation and Hydroxylation at Specific Pro, Gln, Phe, and Tyr Residues Lead to Reduction and Modification of Celiac-Toxic Peptides

Considering that the principal toxic components were not randomly scattered but clustered in Pro- and Gln-rich regions of gliadin, the modification and degradation of celiac-toxic peptides with application of CJAP plasma became our principle objective. Free radicals are available to attack substrates in various media, and the •OH accessibility of Pro, Gln, Phe and Tyr residues is critical for the formation of oxidation and hydroxylation products under CJAP plasma conditions. The higher Pro content in peptide PQPQLPY than in peptide QQPFP resulted in larger amounts of 2-pyrolidone products under RONS attack. A larger number of hydroxylation products was found in PQPQLPY than in QQPFP (Supplementary Figure S2). This finding may be attributed to the location of Tyr residue at the C-terminus of PQPQLPY, which was more attractive for OH radical attack [30]. Besides, the hydroxyl group in the benzene ring of Tyr residue could also increase reactivity of ortho-/meta-carbon compared with that of Phe residue.

The peak area of P1~P7 first appeared to increase, possibly owing to bond cleavage in peptide side chain, which was due to the addition of an oxygen atom to alpha carbon to generate a carbonyl and the introduction of a hydroxyl group resulting from the addition reaction on an aromatic ring of Phe [31,32]. Importantly, the P1, P2 and P6 detected by LC-MS/MS indicated that •OH-mediated cleavage of the peptide played an important role in attack of Pro residue at the C-terminus of specific pentapeptide and heptapeptide in 2-pyrrolidone formation in the presence of O_2_ [33]. For P3 and P4, •OH rapidly attacked the benzene ring of Phe to generate a hydroxyl cyclohexadienyl radical, followed by a reaction with O_2_ and subsequent elimination of •OOH to form *o*-/*m*-/*p*-Tyr (P3) and DOPA stereoisomers (P4). The substituent containing an unshared electron pair on the benzene ring can form a *p*-π-conjugated system with the benzene ring, resulting in an increase in the electron cloud density of the benzene ring, especially at ortho and para positions, which favors attack of the electrophilic group •OH generated by the plasma treatment process to form a mixture of *o*-/*m*-/*p*-Tyr (P3), which was responsible for three peaks in extract ion chromatograms as shown in Supplementary Figure S3A [34]. Attack of the second hydroxyl group has more positional selectivity due to the strong guiding action of the first hydroxyl substituent, leading to generation of the DOPA products (P4) [21].

Backbone cleavage mediated by the highly accumulated OH radical population induced by CJAP-plasma appears to occur at multiple sites, with positions adjacent to Pro residues at the C-terminus being of major importance [35]. With an open structure and water-soluble properties, two model peptides show greater availability for attack by OH radicals. Encouragingly, plasma treatment of peptides was more efficient in terms of structural changes of peptide than Fe/EDTA/AA treatment of QQPFP, for which 22% of the original peptide remained intact after 24 h of oxidation [5]. In contrast, 0.16% of peptide remained after 60 min of plasma processing in our experiment.

### 3.3. Decreased Immunoreactivity of Wheat Gliadins Modified by the CJAP Plasma

The chief epitope for R5 antibody binding is specific pentapeptide (QQPFP) in gliadins. Modification of the FP motif was a key factor in reducing the immunoreactivity of gliadin in R5-antibody recognition [30]. The Pro and ring structure of Phe can be oxidized more readily by OH radicals generated by CJAP plasma in our work. Numerous Pro and Gln are present in the primary structure of gliadin, and smaller fragments of gliadins could be produced at the α-carbon side of Pro and Gln when it was attacked by the RONS induced by CJAP plasma, making it easier for OH radicals to approach the exposed Pro sites of gliadins. Considering the fragmentation and side-chain modifications of the gliadin epitopes, a decreased ability of R5 antibody for detection of its binding sites was achieved in vitro. The 48.05% of original immunoreactivity against the R5 antibody was retained in gliadin treated with plasma for 60 min, compared with approximately 60% following 2-h metal-catalyzed oxidation of hordein [5]. The CJAP plasma could bring about the backbone cleavage and degradation of gluten protein (Figure 11), which resulted in the exposure of more epitope for •OH. Besides, it could produce the higher density of OH radicals at high voltage. These both contribute to the more decreased immunoreactivity than that of metal catalysis.

It is worth mentioning that although we have proved that CJAP plasma can reduce the immune activity of gliadin in vitro, more studies need to be done to verify the effectiveness of cold plasma technology. The gluten-specific CD4^+^ T-cells could be activated inappropriately, triggered by gluten peptides bound to DQ2 and DQ8 heterodimers in vivo [4]. To date, 24 celiac disease-related epitopes have been identified from wheat gluten that induced T-cell response in patients with celiac disease [36]. Hence, it is important that using specific T-cell clones to evaluate celiac immunological activity [37]. Furthermore, it is reported that epitopes rich in Glu and Pro are derived from gluten across all different classes and the celiac disease is triggered by very small amounts of gluten [38]. Therefore, optimization of the discharge parameters of cold plasma is necessary to improve the efficiency of decreasing the immune activity. The development of stronger energy plasma could encourage to eliminate the celiac sensitivity of gluten in the future. Regarding that gluten is embed in complex matrix of lipids, starch and proteins, the region exposed to the digestive enzymes is closely related to those matrices. This accounts for the problematic quantification and identification of digestible peptides, which is significant for the gluten epitopes related to celiac disease [39]. Plasma modification target should be extended to the complex and diverse foods in the practical application.

### 3.4. Decrease in Large-Sized Polymers through Longer Exposure to CJAP Plasma

The agglomeration state with reduction of large-sized proteins and the appearance of small-sized proteins were observed by SDS-PAGE and SE-HPLC. We attributed the increase of small-sized aggregates to bond cleavage at Pro and Gln of the original protein due to the oxidation of RONS generated by CJAP plasma [40]. Based on the LC-MS results, it was found that plasma modification could cause quantitative decrease in gliadin with selectivity for γ-gliadin, and the longer plasma treatment, the greater was degree of protein reduction (Figure 8). The N-terminus comprising mostly highly repetitive sequences rich in Pro and Gln is distinct for each type of gliadin. For γ-gliadin, the typical unit, QPQQPFP, is repeated up to 16 times, which is more than that of the other gliadins [41]. γ-gliadin contained lower single-bond energy amino acid residues and its arrangement was less regular and less compact than that of α/β gliadin, which could also facilitate its attack by RONS [30,42].

### 3.5. Proposed Mechanism of the Model Peptide and Wheat Storage Protein Modified by CJAP Plasma

**Figure 12:**
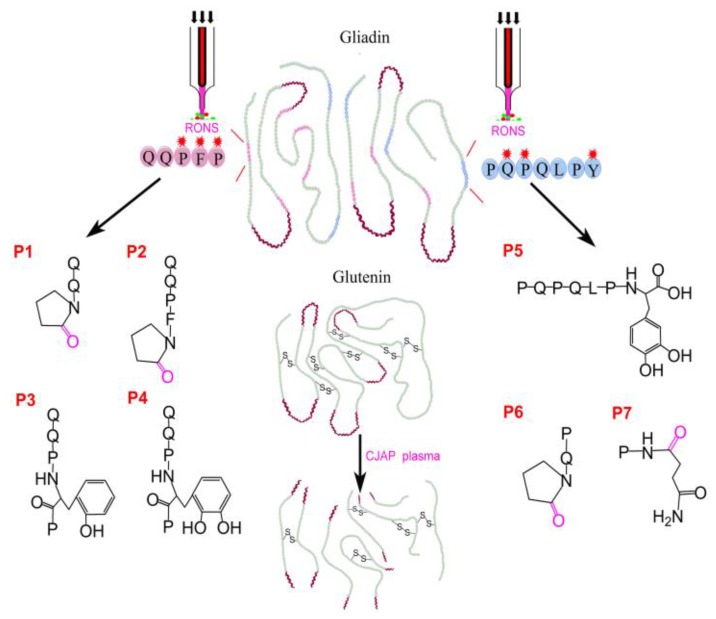
Schematic mechanism for wheat storage proteins and model celiac-toxic peptides modified by CJAP plasma.

Based on the analytical results described above, possible mechanisms of two model peptide and wheat storage protein modified by CJAP plasma are shown in Figure 12. By performing cold jet plasma treatment under atmospheric pressure, large quantities of RONS could be produced. These RONS could cause the depolymerization of gluten protein and quickly attacked specific Pro and Gln residues to give rise to the 2-pyrolidone (P1, P2 and P6) and alkoxylate products (P7), respectively. In addition, aromatic amino acids such as Phe and Tyr were hydroxylated, and monohydroxylated Phe (P3), DOPA (P4) and monohydroxylated Tyr (P5) products are formed in the presence of RONS. Amino acid modifications and peptide cleavage, incorporated with gluten protein fragments induced by plasma treatment, generated a variety of smaller-sized aggregates and reduced the immunoreactivity of wheat storage protein.

## 4. Materials and Methods

### 4.1. Materials

The celiac-toxic peptides QQPFP (molecular weight, MW = 615.68 g/mol, purity 85.1%) and PQPQLPY (MW = 841.95 g/mol, purity 89.5%) were synthesized by Bioyeargene Biotechnology Inc. (Wuhan, China). The gliadins and storage proteins in wheat were extracted from flour of Zhengmai 9023 (*Triticum aestivum* L.), which was planted in the experimental field at Huazhong University of Science and Technology (HUST) (Wuhan, China).

### 4.2. Discharge, Current and Optical Emission Spectra (OES) Detection

Set voltage and discharge current were simultaneously measured by a Tektronix MSO 3045 mixed signal oscilloscope with a Tektronix P6015A high-voltage (H.V.) probe and a Pearson current monitor (Pearson Electronics, Palo Alto, CA, USA).

### 4.3. Extraction of Gliadins from Wheat

The gliadins in wheat were extracted as described by Marsh (2000) [42]. A fraction of 100 g flour was added to 1 L of water-saturated butanol solution and stirred constantly for 1 h at 20 °C After centrifugation (5000× *g*, 10 min, 20 °C) the precipitate was mixed with 1 L of 0.5 M NaCl for 1 h at 20 °C. This step was repeated twice. Next, pellet was washed with 1 L of distilled water for 10 min and centrifuged (5000× *g*, 10 min, 20 °C) again. The precipitate was dissolved in 1 L of 70% ethanol (*v*/*v*) for 1 h at 20 °C and centrifuged at 50,000× *g* in 20 °C for 10 min to collect supernatant. The supernatant containing gliadins was obtained during this procedure.

### 4.4. O_3_ Measurement

The content of O_3_ was detected using N,N-diethyl-p-phenyl-enediamine (DPD) method. A commercial kit (HuanKai Biotechnology, Guangzhou, China) was used in this experiment. Treated samples were supplemented with DPD reagent, which could react with O_3_ to induce a color change of solution. The color depth of solution is positively correlated with amount of O_3_.

### 4.5. H_2_O_2_ and pH Measurement

The concentration of H_2_O_2_ was measured using a Hydrogen Peroxide assay kit (Beyotime, Shanghai, China). The testing solution was mixed with sample at a ratio of 1:1 (*v*/*v*). 200 μL of mixed solution was allowed to remain at room temperature for 30 min and measured at a wavelength of 570 nm using microplate reader. pH test strips were used to measure the pH value.

### 4.6. •OH Measurement

The terephthalic acid (TA) method was employed for determination of hydrogen groups. TA was dissolved in 1.4 mM NaOH solution to obtain a 0.2 mM TA solution. The fluorescence microplate reader (FlexStation3, Molecular Devices, San Francisco, CA, USA) was used to measure the absorbance value of hydroxyl group at an excitation wavelength of 310 nm and an emission wavelength of 425 nm.

### 4.7. Total Content of NO_2_^−^ and NO_3_^−^ Determination

The total concentrations of NO_2_^−^ and NO_3_^−^ were analyzed with Nitric Oxide (NO) assay kit (Nitrate reductase method, Nanjing Jiancheng Bioengineering Institute, Nanjing, China). The reaction between NO_3_^−^ and nitrate reductase leads to the formation of NO_2_^−^.

### 4.8. Identification of CJAP Plasma Treatment Fragments by Orbitrap HR-LC-MS/MS

The Q. Exactive Orbitrap HR-LC-MS/MS was applied to analyze the obtained CJAP plasma treatment peptide mixtures. An Ultimate 3000 UPLC system equipped with an Accucore aQ column (2.1 × 150 mm, 2.6 μm Thermo Scientific, Waltham, MA, USA) was coupled to the quadrupole electrostatic field orbital trap mass spectrometer (Q Exactive, Thermo Scientific, Waltham, MA, USA) in positive mode. The ESI pattern was used as ion source with a scanning range from 50 to 2400 *m*/*z*. Solvent A and solvent B consisted of an aqueous solution of 0.1% formic acid in water and of 0.1% formic acid in acetonitrile, respectively. A gradient of acetonitrile from 99% solvent A to 99% solvent B was generated for 20 min at a flow rate of 0.2 mL/min. The Xcalibur software (Thermo Scientific, Waltham, MA, USA) was applied for analysis of MS result.

### 4.9. Quantitative 2-DE and LC-MS Analysis

The CJAP plasma-treated wheat gliadins were separated by 2-DE as described by Nagib et al. (2010) [43]. First, 200 μL of the gliadin sample (1.25 μg/μL) was mixed with hydration loading buffer containing 8 M urea, 0.2% Bio-Lyte (*w*/*v*), 4% CHAPS (*w*/*v*) and 65 mM dithiothreitol (DTT). Each sample was subjected to IPG prefabricated strips pH 3–10 (7 cm, ReadyStrip, Bio-Rad, Irvine, CA, USA) with 16 h rehydration. IEF was applied using a PROTEAN IEF Cell (Bio-Rad, Irvine, CA, USA) at 17 °C with a series of voltages: 250 V and 500 V for desalting, 500–4000 V linear boost and 4000 V for focus. After IEF, IPG prefabricated strips were equilibrated in an equilibration buffer containing 6 M urea, 2% SDS (*w*/*v*), 0.375 M Tris-HCl (pH 8.8) and 20% (*v*/*v*) glycerol including 2% (*w*/*v*) DTT for 15 min, followed by a strip equilibration buffer containing 2.5% iodoacetamide (*w*/*v*) for another 15 min. In the second dimension, SDS-PAGE of a 12% separation gel at 20 mA was applied until blue dye line reached the end of gel. 

Three sets of selected protein spots from 2-D gels were excised for trypsin digestion (37 °C, 16 h), according to the literature reported by Martinez-Esteso et al. (2016) [44]. The obtained peptide samples from digested protein spots were dissolved (0.1% formic acid, 5% acetonitrile) and loaded into the Q Exactive mass spectrometer equipped with a 75 μm i.d. × 150 mm, packed with an Acclaim PepMap RSLC C18, 2 µm, 100 Å, nanoViper column for sequence identification (Thermo Scientific, Waltham, MA, USA). Solvent A and solvent B consisted of an aqueous solution of 0.1% formic acid in water and of 0.1% formic acid, 80% ACN, respectively. The MS original file was processed and converted using MM File Conversion software to obtain the MGF format file, and the NCBI database and UniProt sapiens database were retrieved using MASCOT.

### 4.10. Qualitative and Quantitative Detection of Carbonyl Groups

The content of carbonyl groups was measured by 2,4-dinitrophenyl -hydrazine (DNPH) method [24]. First, 50-μL gliadin sample was supplemented with 800 μL of 10 mM DNPH in 2.5 M HCl, with 2.5 M HCl as a control. Two tubes were placed at room temperature for 1 h. The samples were precipitated with 1 mL of 20% trichloroacetic acid (TCA) (*w*/*v*) and left in an ice box for 5 min. After centrifugation (10,000× *g*, 10 min, 4 °C), the precipitate was washed with 1 mL of 10% TCA and centrifuged (10,000× *g*, 10 min, 4 °C). The precipitates were washed three times with 1 mL ethanol-ethyl acetate (1:1, *v*/*v*). The obtained pellets were dissolved in 500 μL of 6 M guaninide hydrochloride with general vortexing and again centrifuged (10,000× *g*, 10 min, 4 °C). Finally, 220 μL of supernatant from each tube was collected and the carbonyl content was calculated based on the absorption at 375 nm.

### 4.11. Sandwich Enzyme-Linked Immunosorbent Assay (ELISA)

A commercial ELISA kit (Gliadin, ml058393-2, Shanghai Enzyme-linked Biotechnology Co. Ltd., Shanghai, China) was applied to measure the concentration of prolamin. In the first step, 10 μL of CJAP-treated and untreated samples were added to 40 μL of sample diluent. All samples were then incubated at 37 °C for 30 min. The samples were washed five times with a washing solution. The samples were mixed with 50 μL of HRP-Conjugate reagent, incubated again at 37 °C for 30 min and then incubated and washed as described above. Thereafter, 50 μL each of reagent A and reagent B were mixed in the dark at 37 °C for 10 min. Finally, 50 μL of stop solution was used to terminate reaction. The concentration of prolamin can be calculated by absorption curve measured at a wavelength of 450 nm. Samples without addition of HRP-Conjugate reagents served as a blank control.

### 4.12. SE-HPLC Analysis

The gluten proteins were collected with 50 mM of sodium phosphate buffer with 0.5% SDS (*w*/*v*, pH 6.9) from flour of Zhengmai 9023 (*T. aestivum* L.), based on the documentation reported by Li et al. (2017) [45]. A 20-μL portion of supernatant filtered from a nylon membrane (pore size 0.45 μm) was poured into a Waters 1525 binary HPLC pump, fractionated on a Phenomenex Biosep-SEC-s4000 column (20 min, 0.5 mL min^−1^) and then further tested at 214 nm using a Waters 2998 photodiode array detector (Waters Corp., Milford, CT, USA).

### 4.13. Statistical Analysis

The statistical software GraphPad Prism 6.0 (Microsoft Corporation, Redmond, WA, USA) was used for statistical analysis via one-way analysis of variance (ANOVA) followed by Tukey mean-comparison procedure. The 5% significant differences (*p* < 0.05) and 1% highly significant differences (*p* < 0.01) were evaluated using Origin 8.1 (OriginLab Corporation, Northampton, MA, USA).

## 5. Conclusions

In conclusions, fragmentation at Pro residues, along with modifications at Phe and Tyr residues of two CD-related peptides—QQPFP and PQPQLPY—could be achieved with the application of CAJP plasma. A variety of RONS such as OH, O_3_, H_2_O_2_, NO_2_^−^, and NO_3_^−^ were observed to increase during plasma processing. In addition, the immunoreactivity of gliadin using the R5 antibody appeared to be reduced because a large amount of recognition epitopes were modified after plasma treatment. Furthermore, the longer the plasma treatment, the greater was the formation of carbonyl and hydroxylation products and, consequently, the smaller was the size of the formed aggregates. The structural changes in the two model peptides, as well as the quantitative changes in gliadin after plasma treatment, favored the preparation of gluten-related product for the celiac disease patients.

## Figures and Tables

**Table 1:** LC-MS identification of wheat gliadin spots in 2-DE. The protein name, accession number, mass, pI, protein score, protein match, protein coverage, peptide sequence and mass error (ppm) were presented.

Spot	G1	G2	G3
Predominant protein	Gamma gliadin	Gamma gliadin	Gamma gliadin
Accession number	F2XAR5	Q30DX7	K7WVC4
MW	37,564	34,502	33,067
pI	8.51	8.57	8.88
Protein score	94	1762	2831
Protein match	2	91 (54)	62 (52)
Protein coverage	-	25.5	15.8
Sequence	R.APFASIVAGIGGQ. (ppm: 5.18) R.SLVLQTLPSMCSVYVPPECSIMR.A (ppm: −44.56)	R.TTTSVPFGVGTGVGAY. (ppm: 8.86) R.ILPTMCSVNVPLYR.T (ppm: 6.64) R.ILPTMCSVNVPLYR.T (ppm: 6.85) K.VFLQQQCSPVAMPQR.L (ppm: 9.19) R.SQMLQQSSCHVMQQQCCQQLPQIPQQSR.Y (ppm: 8.80)	R.SDCQVMQQQCCQQLAQIPR.Q (ppm: 6.96) R.SDCQVMQQQCCQQLAQIPR.Q (ppm: 0.35) R.QPQQPFYQQPQQTFPQPQQAFPHQPK.Q (ppm: 5.81)

The UniProt database was utilized to the spectral datasets searching.

## Supplementary Materials

Supplementary materials can be found at https://www.mdpi.com/1422-0067/21/3/1012/s1. Figure S1. HR-LC chromatogram analysis of CJAP plasma-modified QQPFP and PQPQLPY identified by Orbitrap HR-LC-MS/MS at treatment times of 0 min, 5 min, 10 min, 30 min and 60 min, respectively. (A) QQPFP, (B) PQPQLPY. The black arrow indicates CJAP plasma-modified products of two celiac-toxic peptides. Figure S2. Two model peptides QQPFP and PQPQLPY modified by CJAP plasma. (A) The changes in the HR-LC-MS/MS peak area of QQPFP and PQPQLPY with the different treatment time. (B) The changes in HR-LC-MS/MS peak area of products P1~P4 from QQPFP with the different treatment time. (C) The changes in HR-LC-MS/MS peak area of products P5~P7 from PQPQLPY with different treatment time. Figure S3. Extract ion chromatograms (EIC) selected for certain fragmentation ions were utilized to certify the intensity changes of P3 (A), P4 (B) and P5 (C). Figure S4. SDS–PAGE analysis of the gliadins in wheat with different CJAP plasma treatment times. M: Protein molar mass standard, lane 1: 0 min treatment, lane 2: 5 min treatment, lane 3: 10 min treatment, lane 4: 30 min treatment and lane 5: 60 min treatment.

## Abbreviations

CJAP	cold jet atmospheric pressure plasma
RONS	reactive oxygen and nitrogen species
CD	celiac disease
QQPFP	Gln-Gln-Pro-Phe-Pro
PQPQLPY	Pro-Gln-Pro-Gln-Leu-Pro-Tyr
MW	molecular weight
kDa	kiloDalton
SDS	Sodium dodecyl sulfate
DNPH 2	4-dinitrophenyl-hydrazine
EDTA	ethylenediamine-tetraacetic acid
DTT	dithiothreitol
AC	alternating current
OES	optical emission spectra
H.V.	high-voltage
DPD	N,N-diethyl-p-phenyl-enediamine
TA	terephthalic acid
HR-LC-MS/MS	high resolution-liquid chromatography-mass spectrometry/mass spectrometry
EIC	extract ion chromatograms
SDS-PAGE	sodium dodecyl sulfate-polyacrylamide gel electrophoresis
2-DE	two dimensional electrophoresis
IEF	isoelectric focusing
ELISA	sandwich enzyme-linked immunosorbent assay
SE-HPLC	size exclusion high performance liquid phase chromatography
HMW	high molecular weight; LMW, low-molecular-weight
