# Supplementary material for: Effects of Cold Jet Atmospheric Pressure Plasma on the Structural Characteristics and Immunoreactivity of Celiac-Toxic Peptides and Wheat Storage Proteins
Source: Int J Mol Sci. 2020 Feb 4;21(3):1012. doi: 10.3390/ijms21031012 (PMC7036898; doi:10.3390/ijms21031012)
Supplement: Supplementary file 1 [file ijms-21-01012-s001.pdf]

Effects of cold jet atmospheric pressure plasma on the structural characteristics and immunoreactivity of celiac-toxic peptides and wheat storage proteins

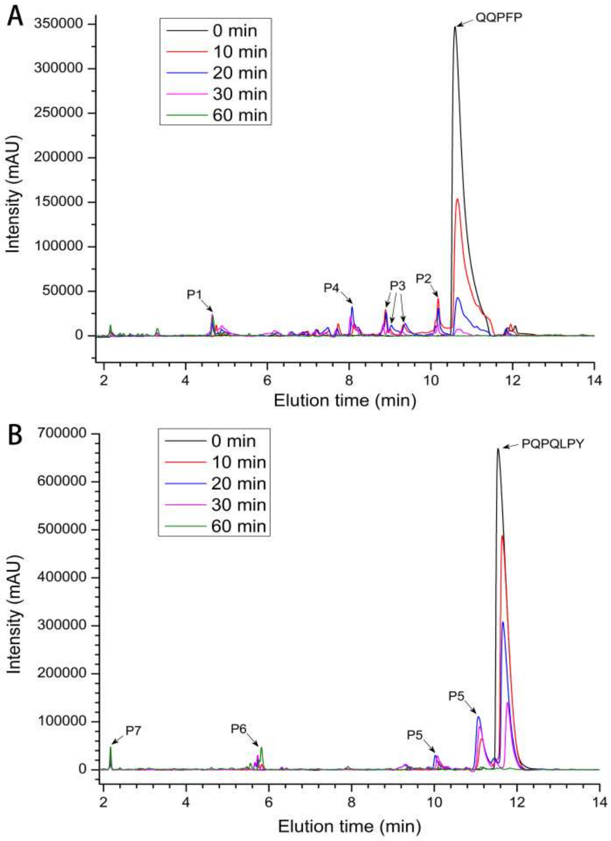

Supplementary Figure S1. HR-LC chromatogram analysis of CJAP plasma-modified QQPFP and PQPQLPY identified by Orbitrap HR-LC-MS/MS at treatment times of 0 min, 5 min, 10 min, 30 min and 60 min, respectively. (A) QQPFP, (B) PQPQLPY. The black arrow indicates CJAP plasma-modified products of two celiac-toxic peptides.

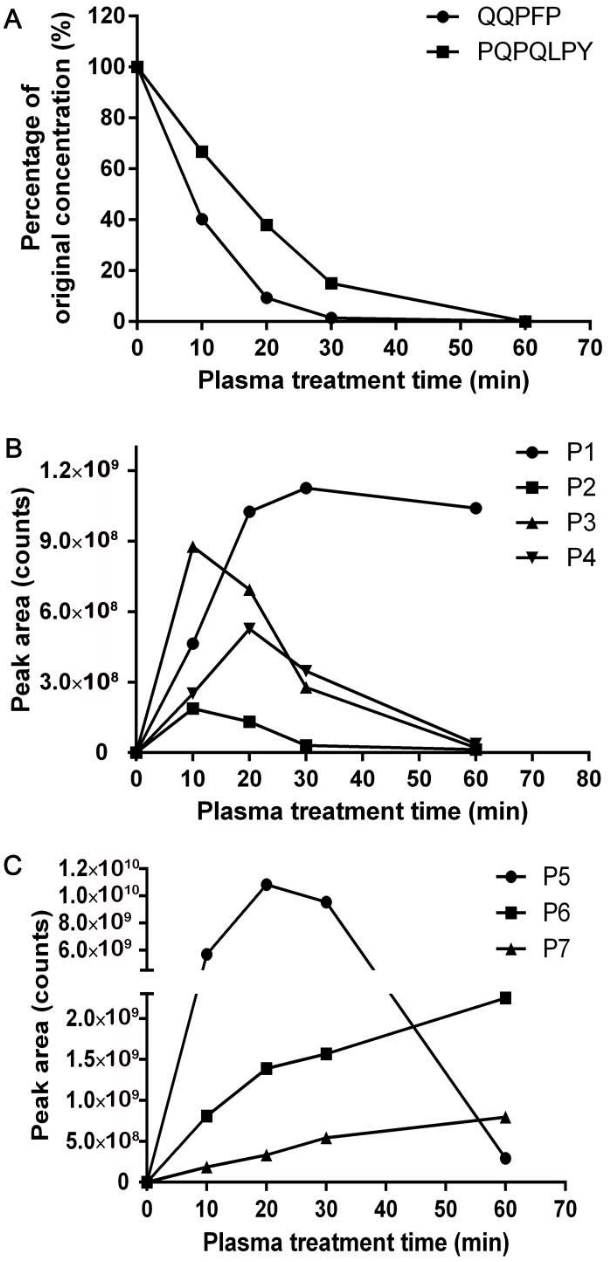

Supplementary Figure S2. Two model peptides QQPFP and PQPQLPY modified by CJAP plasma. Supplementary Figure S1. Two model peptides QQPFP and PQPQLPY modified by CJAP plasma. (A) The changes in the HR-LC-MS/MS peak area of QQPFP and PQPQLPY with the different treatment time. (B) The changes in the HR-LC-MS/MS peak area of products P1~P4 from QQPFP with the different treatment time. (C) The changes in the HR-LC-MS/MS peak area of products P5~P7 from PQPQLPY with different treatment time.

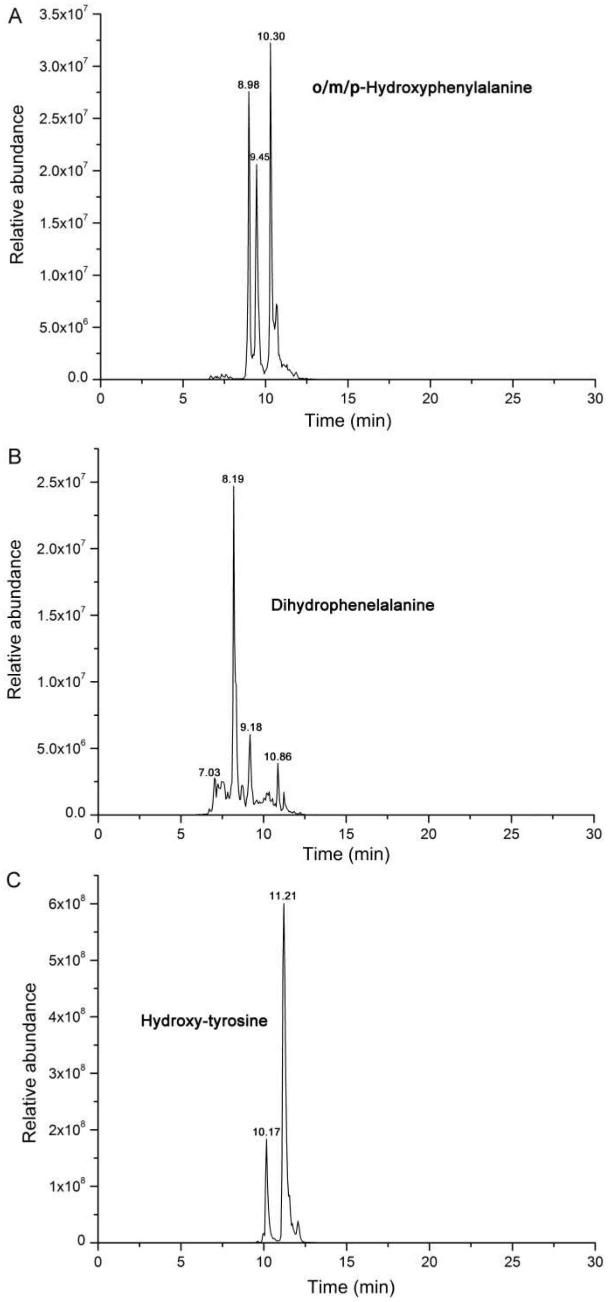

Supplementary Figure S3. Extract ion chromatograms (EIC) selected for certain fragmentation ions were utilized to certify the intensity changes of P3 (A), P4 (B) and P5 (C).

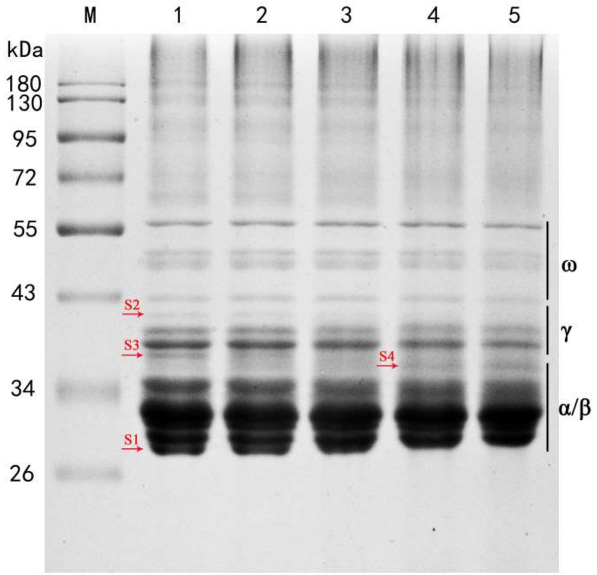

Supplementary Figure S4. SDS–PAGE analysis of the gliadins in wheat with different CJAP plasma treatment times. M: Protein molar mass standard, lane 1: 0 min treatment, lane 2: 5 min treatment, lane 3: 10 min treatment, lane 4: 30 min treatment and lane 5: 60 min treatment.
